# Book Reviews

**Published:** 1816-08

**Authors:** 


### CRITICAL ANALYSIS

### *OF RECENT PUBLICATIONS*

IN THE

### DIFFERENT BRANCHES OF PHYSIC, SURGERY, AND MEDICAL PHILOSOPHY.

Scimus et hanc veniam petimusque damusque vicissem.

DR. DICKSON'S *Remarks in Reply to the Review in the last Medical and Physical Journal.*

THE very favourable terms in which a paper of mine on the Utility of Blood-letting and Purgatives in Fever is mentioned in the last Number of this Journal, would naturally indispose me to offer any other than explanatory remarks in return, did I not, at any rate, feel a great aversion to every thing resembling controversy. My present object, therefore, is not to advert to any difference of opinion between the Reviewer and myself, further than it may appear to have been founded on misapprehension, on either side; and I am happy to take the same opportunity to express my wish to be considered one of those—open, I hope, to conviction, yet when silence, as it may proceed more from disinclination than from inability to reply, does not necessarily imply assent.

I must, however, in the first place, deviate from confining myself to explanation, by unwillingly noticing the remark on "the unfortunate influence of slang," as applied to the language of my predecessors. I cannot, indeed, judge as to its being adopted "to avoid a coarser word," as really none more so occurs to me at present, for "in such language I am little skilled." How far it is applicable to the following passage, within inverted commas, I must leave for others to determine: it was reported to me to be "synochus, frequently terminating in typhus, and death, if copious evacuations had not been had recourse to at an early period of the disease."

I think the gentleman who made this report might fairly challenge the objector to produce a sentence by which the then state of disease he meant to pourtray could be better described, or in so few words, independently of the value of the practical conclusion. It is, however, so nearly in the language of Dr. Cullen's definition of synochus,—"initio synocha, progressu et versus finem typhus,"—as to leave no doubt that the objection is not pointed at the reporter, but against nosology in general, in which I can by no means agree; and particularly against the system of that nosologist who (though no one thinks of defending his theoretical speculations at the present day) was once considered, and, by many, is still considered, one of the brightest ornaments of that school of medicine, for which I entertain a very high respect.

I do not see any great inconsistency in a disease being "at once *mitior* and *lethalis*," alluding to the remark of Dr. Haygarth;—the comparative being wholly relative, its degree of severity must be graduated by a reference to the severest form of the disease, and therefore, though it must be milder than that, it may be still sufficiently severe to prove fatal: at any rate, there can be no doubt of the fact, as the term has been applied here. To this superfluous remark it is scarcely necessary to add, that the quotation was introduced simply to exemplify and give weight to a very requisite caution as to the fatal tendency of a disease if not controlled, against which the mere description of the ordinary symptoms might not put us sufficiently upon our guard.

As the Reviewer considers it a happy opportunity to contrast some opinions of mine on the doctrine of contagion with those of the writer of the article preceding mine, in the Edinburgh Medical and Surgical Journal, he must have misunderstood one of us; for on that head no difference of opinion appears. That ingenious writer, according to his own shewing, was treating of "a totally different disease," when "such a radical difference of character argues a corresponding difference of causation."

I have ever lamented and opposed the term *typhus icterodes* as applied to the West-India epidemic: in a word, I was treating of a fever which I am of opinion is,—He, of the yellow fever, which I am of opinion is *not*, contagious. The peculiar causes to which he alludes, "where all are equally exposed," are those of climate and locality, owing their very existence to a tropical sun; and, therefore, can have no reference whatever to causes operating in the river Medway, in the middle of winter.

To the distinction that has been made between "infection and contagion," to which the reviewer, with better reason, next adverts, I have not been inattentive; nor, in appearing to use these terms indiscriminately, was I swayed by the example of so many of our best authors, who have done so before me, but by the difficulty, and often the impossibility, of ascertaining, where there is a concurrence of two or more causes, to which, or to what share of each, the effect is to be imputed. If, according to one of the first philosophers of the present day, the science of medicine be not yet sufficiently advanced to allow us to obtain just theories from reasoning by the method of induction,—that this remark can, in none of its branches, be more applicable than to the imperfect state of our œtiological knowledge, will sufficiently appear by the following, which has been considered the best explanation of the difficulties of the inductive process that has been given:—"As we can, in no instance, perceive the link by which two successive events are connected, so as to deduce, by any reasoning, *à priori*, the one from the other, as a consequence or effect, it follows that, when we see an event take place which has been preceded by a combination of different circumstances, it is impossible for human sagacity to ascertain whether the effect is connected with *all* the circumstances, or only with a part of them; and (in the latter supposition) which of the circumstances is essential to the result, and which are mere accidental accessories, or concomitants." Stewart's Philosophy, p. 320.

The only way of coming at the truth (as the author then proceeds to shew) is by leaving out all the different circumstances successively, and observing with what particular combination of them the effect is conjoined,—or, synthetically, by combining all the various circumstances,—this, it will be allowed, is but very little and partially practicable in pathological investigations.

The reasoning of the Reviewer as to the danger from foul air generated in a hospital ship, crowded with sick, to all who approached within the sphere of its influence, may apply to those persons seized in, or from, visiting these establishments,—a very small proportion indeed; but it is evidently less applicable to other ships, particularly with a reduced complement of men, after being relieved of their sick, and after the institution of preventive measures; and still less so in individual examples; for, though I am anxious to allow every possible credit to these measures in mitigating fever, I might instance its longest continuance in, unquestionably, one of the cleanest and best ventilated ships of the squadron. This, however, may be better exemplified by a reference to our own fleet, where preventive measures were carried to a much higher pitch than was here practicable, from the season and the difference between the two services. After the the action of the 1st of June, a fever was communicated to two-thirds of our fleet, by intercourse with the French prisoners, and the prizes, which were sickly; and it was not until after 800 men had been sent to the hospital, that the ships were considered fit for sea, towards the end of July; although Dr. Trotter shows that the highest possible attention was paid to preventive means, "so that not a particle of impure air could lodge any where," "and not even the slightest cases were allowed to remain in the ship." Med. Naut. vol. i. p. 80–81. In such, and in numerous similar instances recorded, as well as in some of those in the Medway, (where I was particularly struck with the applicability of a remark of Dr. Wilson Philips, when treating of febrile contagion, which I shall subjoin,) the attempt to explain the prolongation of the disease by crowding, impure air, or other causes, "dissimilar to their effects, for that, I fancy, is the distinction alluded to, would be evidently unsatisfactory, without the admission of farther and specific agency: "It is a curious and wholly unaccountable fact, (says Dr. W. P.) that contagious diseases generally run a certain course, notwithstanding all the means that can be employed to check them, and after this cease spontaneously, while the walls, furniture, &c. must still be supposed to be highly impregnated with the contagion. This is observed by Russel and others in the plague, and in all contagious diseases."

Any farther remarks, (for, as the Reviewer has not been very explicit on this head, these may not be strictly relevant,) I shall beg leave to defer, at least until I have been favoured with the perusal of the Dissertation on Contagion alluded to; but that I have not been unaware of, nor inattentive to, the distinction in question, nor to the vast import of precision in medical language, will appear by a Report on the Yellow Fever, previously written, though not yet published, in which I have not only adverted to the distinction between contagious and infectious disorders, but suggested the expediency of adding a 3d class, possessing no native power of communication, which, from the want of a better term at the time, I called diffusible diseases; the final syllable implying merely the possibility, under a peculiar coincidence of circumstances, of their diffusing themselves; not that they are either naturally, or, under ordinary circumstances, capable of being so diffused.

Setting this aside, the whole objection of the Reviewer seems to consist in my not having discarded "that unfortunate word typhus." This, certainly, was not entirely optional tional with me in giving the opinions of others as well as my own; nor has he shown how I could have done so, had I been inclined, without mutilating, and, perhaps, perverting, the meaning of the reports with which I had been favoured. Nay, the very object of my paper being to prove the utility of bleeding and purging in a fever which, when not controlled, assumed the character so well known under the name of typhus (for I was not aware that this practice "is now pretty universally admitted, in many fevers, from whatever cause;") my purpose would, in a great measure, have been defeated by doing so, unless I had employed considerable circumlocution and repetition, to keep in view that I was still talking of a disease universally known under the name I had rejected. I was, therefore, in hopes, by showing, not by theoretical declamation, but practically, and on a very extensive scale, that, instead of debility, putridity, &c. being the consequence, these dreaded results were prevented by the depletory mode of treatment, I had performed a more essential service to practical medicine than by making war upon a word.

With regard to some ingenious enquiries which follow, on the connection between fever and inflammation, being more the offspring of modesty than decision, I am afraid that they may, with greater propriety, be referred to the obscurity of the subject: nor can I perceive that the difficulty is materially diminished by observing that fever "is no more than universally altered action," a definition, with a little modification, applicable to any other disease,—and which appears to me to be saying little more than that in disease there is an altered or different state of action from that in health. For myself, I should be particularly gratified by receiving a more satisfactory explanation than has hitherto been given of the various phenomena of fever; but I am sorry to say, that, to any theory of so complicated and varying a disease, in our present state of knowledge, I feel much more confident of my ability to offer objections, founded on personal observation—than to propose any, that shall be free from objection, in its stead.

*June*, 1816.

We trust Dr. D. will be satisfied that his hint has been attended to; but think it our duty to add, that the gentleman to whom his letter is addressed has been for some months embarked for Jamaica.— Edit.

To

### *To the Editors, &c.*

GENTLEMEN,

Accept my best thanks for your early communication of Dr. Dickson's paper, and for the polite note with which it is accompanied, in which you give me credit for every wish to be candid, and not less to improve our science by rendering its language more perspicuous and correct. I shall now state my defence in as few words as possible. First, it is but fair to give Dr. Dickson credit for his attention to his predecessors. I am ready to acknowledge that, having none of those proofs of their abilities, such as Dr. Dickson has favoured the world with of his own, I was less delicate in remarking what appeared to me to be a dangerous language. As Dr. D. refers us, with much propriety, to Professor Cullen's definitions or characters of synochus, I shall follow him in tracing the writings of the same author in his practical remarks.

"In the case of synocha (says he) there is little doubt about the propriety of blood-letting; but there are other cases of fever, *as the synochus*, in which a violent re-action and phlogistic diathesis appear, and prevail, in some part of the course of the disease [*initio synocha*], while at the same time these circumstances do not constitute the principal part of the disease, nor are to be expected to continue during the whole course of it; and we know that, in many cases, the state of violent re-action is to be succeeded, sooner or later, by a state of debility from the excess of which the danger of the disease is chiefly to arise. It is, therefore, necessary that in many cases blood-letting should be avoided, and, even although during the inflammatory state of the disease it may be proper, the evacuation should not be so large as to increase the state of debility which is to follow."—First Lines, cxl.

Speaking afterwards of purging, he remarks,

"At the same time the evacuation may induce a considerable degree of debility; and, therefore, in those cases in which a dangerous state of debility is likely to occur, purging is to be employed with a great deal of caution, and here the due measure is more difficult to be observed than in blood-letting."—Id. cxlvi.

I am well aware that Dr. Cullen attempts to qualify all this with a number of cautions. But all of them tend to render the practitioner fearful of very bold and free evacuations in an early stage of fever. Nor would a knowledge of the epidemic help him, as the fear of typhus must always haunt him, and often paralyse his efforts. The experience of Dr. Dickson and his predecessors, therefore, taught them to act in direct opposition to Dr. Cullen's cautions; and, instead of apprehending increased debility from early and free evacuations, evacuations, to use them as the means of preventing "typhus and death." After this, why should gentlemen persist in the use of terms which, if applied in the manner directed by the authority on which they rest, must be destructive to the patient. I am ready to admit that the term *slang* might be considered a coarse expression, and further to acknowledge that I wished it to be considered as such, and that its use might prove, if possible, one means of directing the attention of practitioners to the inconvenience arising from nosology, so far at least as relates to acute diseases. In these the symptoms are to be the direction of the practitioner, and a comparison of them, not with Dr. Cullen's nosology or his theories, or his cautions concerning terminations in typhus, but with symptoms which he has seen in other cases, and their relation to morbid appearances, which he has detected in his dissections.

No man has a higher respect for the industry and talents of Dr. Cullen than myself; but it is unreasonable to suppose that one, the extent of whose practice was confined to an inconsiderable district in the north, should be equal to teaching the treatment of acute diseases in camps, fleets, and in tropical regions. In this respect, too, it is pleasing to find some concurrence of sentiment with Dr. Dickson, who "has ever lamented and opposed the term *typhus icterodes;*" his principal objection to which must be the danger of its misleading us in practice. But this is connected with another not less important inquiry, namely, the doctrine of infection.

That the causes of the tropical fever, and of the ship or hospital fever, are different, cannot be questioned; yet if the causes, and even the effects, are different, the mode of producing their effects on the human body may be similar. Scarlatina, small-pox, and measles, are different, in cause and effect, yet, as we know no means by which the effect of either can be produced but by contact or effluvia of a diseased subject to one susceptible of the effects of such effluvia or contact, we do not scruple to impute all to similar, though not to the same causes,—to contagions possessing different properties. I admit there is a still greater difference between the cause of yellow fever and hospital fever, inasmuch as one arises from the state of the atmosphere, unaltered by human effluvia, and only to be induced in a certain temperature; the other, on the contrary, is only to be induced by human effluvia, may exist in almost any temperature, and is usually increased when, on account of cold, the free access of the common atmosphere is excluded. In their mode of producing the disease, they are, however, similar. All who are exposed to the atmosphere of either are endangered. But the the main distinction yet remains. All who receive small-pox, scarlatina, and the other contagions, may excite a similar disease in others, and they in others, and so on as long as those who are susceptible of the impression are exposed to the cause. On the contrary, the yellow fever is known to spread only in tropical heats, and the ship or hospital fever only in a confined atmosphere. During the tropical heat, all exposed to the atmosphere in certain districts are liable to be seized, without coming into contact or within the effluvia of the diseased; and, in a ship or hospital crowded with subjects under fever, all who inhale the air are equally in danger. With the change of atmospheric temperature the cause of yellow fever ceases, and those who expose themselves to the sick are in no danger. Those who only inhale the effluvia of hospitals or prisons, and afterwards sicken and are nursed in a different atmosphere, with care to preserve proper ventilation, are never injurious to others. In both, the concentration of the cause increases the effect. Thus, the tropical fever is, for the most part, greatest when the heat is greatest, and becomes gradually milder as the temperature is milder. The hospital or ship fever gradually lessens in severity as the atmosphere, by a free ventilation, is less impregnated with morbific effluvia. But neither change of temperature, nor a less impregnation of the air with the morbific effluvia, produces any change in the diseases excited by the true contagions. The person exposed to the bed-side of a patient under the worst kind of small-pox, measles, or scarlet fever, has often as mild a disease as one who only receives the disease from the slightest impregnation; from a source which he never could detect. This is enough to show that there are distinctions which it requires nothing more than common observation to detect.

It gives me much pleasure to find that Dr. Dickson has some remarks preparing for the public on Yellow Fever. I have no doubt of his accuracy, and agree with him that it would indeed have been difficult for him to avoid the word typhus. Much of this difficulty I imputed to a laudable, but unnecessary, and, in my opinion, ill-timed modesty, and the paper I am now answering confirms me in that opinion.

Most heartily do I agree with this candid writer, that my definition of fever (universally altered action) "may, with a little modification, be applied to any other disease." The principal modification, indeed, is local and universal. This I conceive the safest of all definitions, as it implies no more than it means, and leaves us afterwards to enquire into the nature of the alteration, or the parts altered.

On the whole, gentlemen, it gives me much pleasure to find so little difference of opinion in so ingenious a writer, and I am persuaded that, for a perfect coincidence, little is wanted but a complete understanding between us.

I have the honour to be, &c.

---

We have collected the following passages to show how often diseases are supposed to be contagious, merely because they happen to be epidemic,—or, to use an expression of which we have before availed ourselves, because they become general from all being exposed to the same cause. In hydrophobia, the case seems reversed. We are apt to impute it to heat, yet, by the above account, the heat of Africa is insufficient to produce the disease, whilst it often is induced from infection in the colder regions.

*Travels in Southern Africa;* by Henry Lichtenstein.

"Symptoms had appeared among the troops of a very malignant epidemic disorder, a sort of dysentery, which had already nearly filled the hospitals at the Cape Town with sick. Most of the patients died in a very short time, under the severest sufferings; and even those who did not die immediately remained with diseases, which, with very few exceptions, proved incurable. Notwithstanding the very great care of the general, that every thing should be furnished which might assist the recovery of the sick, that they should have good wine, good food, proper clothing, and proper covering at night to protect them against the influence of the climate,—notwithstanding all these cares, the evil daily increased. Toward the end of November, there were frequently not less than ten corpses carried out of the hospital in the course of the day. Several excellent officers shared the same lot.

"At length it appeared clear that the situation of the camp was a principal cause of this heavy calamity, since, during the day, it afforded no shelter from the burning heat of the sun, and that it was open to the damp wind which often blows from the sea in the evening, between the two rows of hills, suddenly, as with a breath, dispersing every feeling of warmth, changing the temperature of the atmosphere not unfrequently forty degrees of Fahrenheit's thermometer in the course of an hour. Towards the end of December, therefore, the camp was removed nearer to the Table Mountain, under the shelter of the Wine Hills, as they are called, where, indeed, the ground was less favourable, but the situation was, beyond all comparison, more salubrious. Before this cause of the calamity suggested itself, and the medical practitioners had learnt that an early and plentiful use of mercurial preparations was an infallible specific against the disease, hundreds of our brave warriors had fallen a prey to it. The Waldeck battalion suffered particularly; particularly; for the Germans seemed universally more liable to be seized with the malady than either the Dutch or the natives.*

"I was not spared myself. The absence of most of my colleagues, who were summoned to the town for the service of the hospital, made my post very laborious. As the only surgeon-major in the camp, many times I was called up three or four times in the night to the sick, and, in the day, I had, besides attending eighty patients in my own battalion, many half-sick officers throughout the camp to visit, who naturally wanted so much the more care and attention, even in matters really trifling, in proportion to the generally alarming aspect of the disease. Cooling medicines were indispensible in this painful practice. I found their good effects: by the constant use of them I kept myself for many weeks free from the disease, though under circumstances extremely unfavourable for it. At length, however, I was subdued, and was obliged to quit the camp, and to go into the town. I had, at that time, a patient under my care whose situation lay very heavy on my heart, one of the worthiest of men in every sense of the word, and with whom I lived in the closest friendship. The importance of his post, and his confidence in my skill, had kept him hitherto in the camp, notwithstanding that he was extremely ill, and that I exhorted him very earnestly to go into the town. Now that I was obliged very reluctantly to quit my post, he consented to accompany me, and we took a lodging together, where I could still attend upon him, as far as my strength would admit. Alas! four days after our removal he expired in my arms." Vol. ii. p. 145.

"General Janssens would not now permit me to remain in the house of a stranger, but insisted upon my coming to him. To the very great care and kindness I experienced there, and to the skill of my friend and colleague Von Zinkgraf, added to my own unimpaired youth, am I indebted for having at length gained the victory over my disease. I was for a week in a truly dangerous state. Labouring under the same symptoms which had preceded the death of my never-to-be-forgotten friend, I could not but look upon my own end as fast approaching. My recovery was very tedious, and perhaps the weak state to which I was reduced would have been protracted much longer, if the commissary General De Mist had not taken me to a country-house he inhabited in the Tyger-Mountain, where he and his children took the utmost care of me. The good air I enjoyed there had at length so happy an

* In a note to this passage, the author imputes the disease to a complaint in the liver. Such may be the case, and mercury may have been a specific; but it is greatly to be lamented that the immediate effects of change of position and of mercury are not more precisely marked. One thing, however, is certain, that the disease originated in local circumstances, and ceased its epidemic property as soon as the camp was removed.—Edit.

 effect, effect, that by February I was able to return to the camp and resume my functions." Vol. ii. p. 15.

"It is very remarkable, that no example of madness among the dogs was ever known in the colony; an additional proof that this disease does not so much originate in the temperature of the atmosphere as in other properties belonging to the climate. Perhaps the effect here noticed may be very much ascribed to the nature of the water, which, almost throughout the colony, is strongly impregnated with natron.

"If a murrain prevails among the horses in any place, the most effectual remedy for it is flight; commonly a change of place, though it be only to the distance of a few miles, is sufficient, at the same time to restore the diseased, and to preserve those that continue sound from taking the infection." Vol. ii. p. 33.

---

*Observations and Inquiries into the Nature and Treatment of the Yellow or Bulam Fever, in Jamaica and at Cadiz; particularly in what regards its primary Cause and assigned Contagious Powers: illustrated by Cases and Dissections, with a view to demonstrate that it appears divested of those Qualities assigned to it by Mr. Pym, Sir J. Fellowes, and others. In a Series of Memoirs.* By Edward Doughty, Member of the Royal College of Surgeons in London, and Surgeon to the Forces. 8vo. pp. 239. Highley and Co. London, 1816.

It often happens that men of the most upright intentions, and, in certain points, the best informed, are the least successful in making the most of their case. Though Mr. Doughty stands on very high ground, both as to his opinion of the cause and treatment of yellow fever, yet, for want of a certain habit of arrangement, his book, full of the most conclusive facts, will fatigue those who do not feel a sufficient interest in the questions. Half the facts placed in a stronger point of view, half the arguments well arranged, would have gone further, because the attention of the reader would never have wandered.

The author will not feel offended with these remarks, especially when we add that we have perused his work with much attention, and with no less satisfaction, and that it shall be our endeavour to do him more justice than he has done himself; at the same time advising our readers to procure the original work, as a valuable deposit of most important and well-authenticated facts.

The Preface contains two particulars much too interesting to pass unnoticed. The first is a misunderstanding between Staff-surgeon Doughty and the gentleman "at the head of the the department, with the designation of Director of Hospitals." The origin of this was, a difference of opinion; and, afterwards, a desire of the Staff-surgeon to settle the question by the examination after death of such as had died of the fever. This was only adding to the former presumption; and Mr. Doughty, growing bold from being refused the only means of proof, "in a moment of irritation expressed his sentiments too warmly." The consequence was a temporary dismissal from the service.

We have been particular in this event, because the error is much too common in young men of all professions, and of all others the failing is the most galling to those who, from age or rank, more particularly in any branch of the military, are accustomed to receive a submission prompt, if not servile. It will be asked, Are young men to continue the errors of their predecessors without remonstrance? To this we answer, that the manner of remonstrating is the great difficulty. When left to practise by themselves, and this must often happen, they may act according to their own discretion: if they fail of success, they must alter their conduct, and will not lose their reputation; if their practice prove successful, much management is required in reconciling their superiors to plans and opinions different from their own. But the sure way to prevent reconciliation is by suffering those above them to feel their inferiority. By good management, all may keep on good terms, and the service be infinitely better promoted than by hostilities on either side.

The next remark we have to make is, "the council of trade," meaning, we presume, the board of privy council who take notice of that department, "have instituted inquiries into the [contagious] nature of yellow or Bulam fever, and have directed the medical officers of the navy and army to communicate such information as they may individually possess concerning it." In our opinion, had they required this information of the residents in those countries, whether natives or others, they would have been much more likely to arrive at truth. Men bred in particular schools find it difficult to divest themselves of dogmatas delivered *ex cathædra.* In medicine, we can only reason from fact, or analogically. The former will instruct us in comprehending the nature and cure of diseases; but, till we have acquired that information, which can only be gained by a repeated accumulation of facts, we must trust to analogy in whatever relates to the invasion, cessation, and progress of epidemics. On these subjects, therefore, long residents are by far the best informed; and, if their opinions are often absurd, they will will be best corrected by an examination of the facts on which they are founded. Let us even be cautious how we undervalue their opinions. Had men of real celebrity, among whom we may reckon a Pulteney and a Baker, payed proper attention to the vulgar opinions concerning the cow-pox, how many lives might have been saved before a Jenner became the means, under Providence, of introducing this divine prophylactic! Had the opinions of medical men never been warped by the properties attached to the word typhus, how many charities might have been exercised by the descendants of Great Britain, which were slowly introduced by the French colonists from St. Domingo! During the most distressing epidemic in Philadelphia, when separation and a consequent crowding of the sick was enforced, and when no one could be found who would inspect the care of these devoted wretches, a Frenchman, who had fled from the horrors of anarchy, was the first to offer his assistance. To what can we impute this confidence but to a thorough knowledge of the laws of this epidemic, acquired by a life spent in an island continually ravaged by this dreadful scourge.

This long introduction will save us much trouble, and our readers some time. In the progress of the work we shall say but little concerning the author's opinions, rather producing his facts, by which they may be at once explained and supported. Nor shall we dwell long on the advantage of bleeding, a practice now, we believe, sufficiently established.

The first question, which we were glad to find so soon dismissed, is the dull controversy between Drs. Chisholm and Bancroft on the distinction between the Bulam and yellow fever.—We are almost afraid to introduce "the ship Hankey," lest our readers should throw down the number in despair. We shall, however, offer Mr. Doughty's opinion in a very few words.

"The varieties (says he) pointed out by Mr. Pym, as designated by symptoms and appearances, only evince what I have advanced, that the *Bulam* fever, as it is, in my opinion, inconsistently termed, is only the endemic ardent continued, or yellow fever, of the West Indies, America, Cadiz, and other parts, in its most concentrated or aggravated form.

"The cause from which the several varieties arise, is yet involved in much doubt and obscurity, as is the cause of fever in general, inasmuch as the morbid nature of that cause, whatever it may be, is not cognizable to our senses.

"It is known well, and I invariably observed the occurrence, during the eight years that I held the situation of Assistant, and Regimental

Regimental Surgeon in Jamaica, that, at certain seasons of the year, a peculiar cause does prevail, evinced by its effects; for, although fever is occasionally produced in every month in the year, yet that particular order of it denominated endemic or yellow fever is rarely seen till the autumnal season, viz. from the beginning of August to the latter end of December or January.

"Marsh miasma, which has been ascribed by so many as the cause of fever in all its varieties in the West Indies, does not appear to me to be an opinion by any means conclusive; because the body's exposure to marshy exhalations at certain seasons of the year, in Jamaica, does not produce any derangement of health.

"I am more disposed to agree with an opinion which has been advanced, that, in the extrication of the principles of vegetation, there is imparted from the earth, in particular situations in the West Indies, and in climates of a similar temperature, certain noxious exhalations, which produce effects on the constitution, in a ratio with the age and peculiar susceptibility to receive the morbid impression.

"In the West Indies, from the month of January to July or August, there is generally a continued drought, and during these months, with some occasional exceptions, Europeans enjoy a state of health equal to what they could possibly do in their native climate.

"The 6th battalion of the 60th regiment arrived in Jamaica the beginning of February 1801, upwards of eight hundred strong. They were stationed in Up-Park Camp. From the date of their arrival, to the middle of August, they only lost one man, and he died from drinking to excess of ardent spirits. I joined the battalion as assistant-surgeon shortly after their arrival. I never saw a finer corps both of officers and men.

"From the period of their arrival, as I have stated, to the middle of August, they only lost one man. The season had been uncommonly dry, and every vegetable production around their station was not only parched but burnt up. The rains began to fall, with their usual rapidity, in August, and, before the end of the month, the park, which had been withered and bare the preceding six months, became one continued verdure. Then fevers, in their concentrated form, began to develope themselves amongst the officers and soldiers of the 6th battalion. Dr. Kilgour, surgeon of the battalion, a man of considerable talent, was the first officer who fell a victim to the disease. He died with every symptom as described peculiar to the *Bulam* fever; black vomiting, as the strongest characteristic, I well remember. Dr. Kilgour's death was followed, in the same week, by four others, viz. the first assistant-surgeon, the paymaster, one captain, and one subaltern. It is yet in my memory that black vomit closed the scene in four of the above cases.

"There was a mortality amongst the men in an equal proportion to the officers: in three months we lost near two hundred men. It was the first season I had an opportunity of seeing, to any extent, the disease, although I arrived in Jamaica on the 30th of September, 1800."

As we have promised a succinct view of contagion and epidemics, we shall, at present, make no other observation than that, until we meet with other arguments, we are not prepared to admit that marsh miasma is not a cause of yellow fever. What is meant by the extrication of the principal of vegetation, we pretend not to say; but when to this it is added that, there are "imparted from the earth certain noxious exhalations, which produce effects on the constitution in a ratio," &c. we cannot help asking whether noxious exhalations are not miasmata, for, though the existence of marshes may not always be necessary, yet, in many respects, the invasion of this epidemic is similar to that of marsh agues. With both, the autumn is the trying season, and generally with both the most formidable period is under an ardent sun after rains. It is not improbable that in the tropical regions, parts less exposed to stagnant pools may become deleterious; but it is certain that heat without moisture is insufficient to induce the epidemic in any formidable shape.

The author next gives an account of the progress of the disease in himself at Kingston, in Jamaica. This must, of course, be imperfect; but the perusal will be attended with practical advantages. The only passage we shall select as immediately connected with our present inquiry is the following:

"However, (says Mr. D.) though not an advocate for the administering of mercury as a sole remedy in yellow fever, I yet feel convinced to the accidental circumstance of my constitution being influenced by its action I may ascribe my recovery: every untoward symptom was removed when my mouth became sore, and the re-establishment of my health soon followed."

On account of this illness, the author had few opportunities of seeing the full effect of the fever in the camp till the autumn of 1801; but in the following season he remarks,—

"The mortality during the whole season did not amount to one in ten of that in the preceding year, in a like number of cases, where the mercurial plan of treatment was the course pursued."

"Towards the latter end of December, 1801, (says Mr. D.) the sickness, which had been so great and destructive *the preceding three months*, began to decline, and by the middle of January, 1802, had nearly ceased.

"Having now time to reflect on what I had seen, I began to reason upon the nature of the fever, as far as it had fallen under my observation, and the several modes of treatment which had been so unsuccessfully pursued. I also consulted various authors who had written on Yellow Fever, and I became particularly struck with the opinion of Dr. Jackson.

"From January to the end of July there was a continuance of dry weather, and, during that period, a total cessation from every order of acute disease in the 6th battalion.

"Dr. Kilgour was succeeded in the surgency by Mr. M'Intyre, Assistant-surgeon of the 11th regiment. This gentleman (who lately died in India) laboured under a very impaired state of health, which required his absence from the battalion during the months of August and September. The medical charge of the battalion now devolved upon me.

"The rainy season commenced in August, and vegetation, which had been dormant from the previous drought, quickly followed. I observed particularly its apparent influence in the production of morbid action on the European constitution.

"On the first appearance of verdure in the camp this season, the order of sickness, which then developed itself in the battalion, *was slight affections of the chylopoietic viscera, in the shape of diarrhœa; and, as vegetation advanced, these increased in violence, so as to assume more the dysenteric form. Fevers, however, soon followed, first of the mild remitting order, and progressively advancing to the violent ardent endemic; or, as it is more generally denominated, yellow fever, with all those symptoms which are said to mark, as a distinction, the Bulam fever.*"

In tracing the above history, we see that with the commencement of the rainy weather the first order of complaints are *slight*, and even when fever appears, it is at first of the mild remittent kind, progressively advancing; and, though the rainy season commences in July or August, yet, by the beginning of the last extract, it appears September, October, November, and the early part of December, are the most fatal months,—that is, after the first violent rains have fallen, and the effect of an ardent sun is felt by the exhalations it produces.

On the mode of treatment we say little, but that it was such as we might expect from a man of a vigorous mind and unbiased judgment, at the age we may suppose Mr. Doughty to have been. Yet still we cannot help remarking a disposition to systematic detail, which smells strongly of the school.

"The leading points (we are told) to discriminate in the selection of remedies for the cure of yellow fever, are, the age and constitution of the patient, his habits of life, time and station of residence in the climate, and the indications pointed out by the prevailing symptoms. But I found no consideration so important as attacking the disease in its onset; because, so rapid is it in its progress, and destructive in its consequences, that, if the aid of medicine can avail, it must be had recourse to before those fatal derangements, which so quickly follow an accession of yellow fever, in the brain, stomach, and other parts, have too far advanced. If the morbid action produced by the exciting cause, whatever that may be, in the fevers of the West Indies and Andalusia, is not subdued very early after it has manifested itself, the application of medical powers will be doubtful, or, at least, in a tenfold degree, have less chance of success than when the disease has not impaired greatly the stomach, brain, &c. and when the blood (which it quickly does) has not began to lose its cohesive qualities, and run into a state of putrefaction; pouring into the inflamed stomach in complete solution, producing the striking and invariably fatal symptom—black vomit. For, whatever authors may have said, I believe no man ever recovered if this affection had taken place. I have seen many hundred cases of black vomit, and never knew a patient to survive where the matters thrown up from the stomach had the resemblance of coffee-grounds."

Now, if all this train of symptoms follows with so much rapidity, and often with so much fatality, why should we inquire into the "age, constitution, and habits of life, of the patient?" Are not "the indications pointed out by the symptoms" sufficient? and, if we wait to deliberate on any others, can such deliberation produce any thing but apprehension, delay, and a most dangerous indecision.

We shall now offer an extract of the utmost importance, and which we are anxious should appear in a medical publication, which, like our own, professes to glean improvements from every quarter.

"Since writing the foregoing (says Mr. Doughty) I have accidentally had an interview with Mr. Douglas, formerly a lieutenant in the 85th regiment, whose interesting narrative, as inserted in the Annual Register for 1802, I think very applicable to what I have stated respecting bleeding at the commencement of the disease, more especially as the practice of it, though suggested to Mr. D. by a perusal of Dr. Jackson's Treatise on Fever, was corroborated by Lieutenant (now Major) Franchisen, who had been in the camp with me, and had witnessed the success attending it for two months previous to his embarkation. I therefore insert it here.

"*A true Narrative of the melancholy Situation of his Majesty's Store-ship Chichester, of 44 guns, Captain Stevens, on her Passage from Jamaica to Halifax, Nova-Scotia, in the months of October and November*, 1802 *(never published).*

"'After the Chichester (Captain John Stevens), from England, had delivered her stores at Port Royal, Jamaica, she laid alongside the quay for some time, getting her rigging, yards, sails, &c. examined, examined, or, according to the sea phrase, over-hauled: she was ordered home, and to put in at Halifax. She was very short of her complement of hands, and the 85th regiment, then in the island, being ordered to be reduced to the peace establishment, eighty of the healthiest of the men who were to be discharged, were ordered on board, and embarked on Friday the 8th of October, under the command of Lieutenant Douglas, of that regiment. The ship got out from the quay to an anchoring in the harbour a few days before this, where three of the midshipmen, a sailor, a marine, and a woman, died of a fever. This created some alarm, but it soon vanished on every symptom of that dreadful disease disappearing, and every countenance glowed at the prospect of soon seeing the land of Liberty again.

" 'We weighed anchor, and got out of the harbour on the morning of the 12th. We got clear out from the land that day, and the next morning Lieutenant Miller, first lieutenant of the ship, and several of the ship's crew and of the soldiers, attended the surgeon and his mate, complaining of head-achs, and other symptoms, of an alarming appearance of the yellow fever.

" 'There was very little wind, and that was against us for seven days; we got sight of St. Domingo on Saturday morning the 16th. That night two men and two boys died. We continued tacking between Cape Tiberon and Navasa Island for two or three days, during which the two lieutenants (Miller and Avery), and the only midshipman now left, died, as also four of the 85th regiment, two of the sailors, and two marines. On Wednesday morning a fine favourable breeze sprung up, and upon the Captain being saluted in the morning, according to custom, upon deck, he expressed his regret at the loss he had already suffered in officers and men, and said, "I have lost my two lieutenants, all my midshipmen, and the master is now taken ill; I have hardly any body to trust to the watch, and my men getting, and likely to get, too few for the task that is before them:" and was himself obliged to take to his bed in the afternoon. We passed Cape St. Nicola Mole about eleven o'clock, and in the evening took our departure from Tortuga, a little island on the north-west coast of St. Domingo. It is impossible to describe the distressing sufferings of the sick; nothing could be heard between decks but the most dreadful screeches and howlings of delirious men in the last agonies of death. The medical gentlemen (Surgeon Miller and his mate Mr. Varley) exerted themselves to the utmost of their power, in performing the duties of their profession, and of humanity, to afford every means they could invent for the relief or ease of the distressed. It was particularly recommended to them, before we left Jamaica, to use calomel in every case of the yellow fever: they attended to this in too strict a manner, until they had convincing proofs of its inefficacy.

" 'Mr. Miller had, among his collection of medical books, the treatises of Dr. Jackson on Fevers and other Diseases, which led him, according to that eminent physician's advice, to try bleeding.

" 'The symptoms, from the beginning to the end of this dreadful malady, were such as Dr. Jackson recommends bleeding for. The first trial of this specific was rather as an experiment upon a very desperate case; indeed, Mr. Miller entertained very little hopes of the effect; he was, therefore, averse to it, but was prevailed upon by others to give it a trial, when one of the quarter-masters had been seized with every symptom that hitherto had proved fatal; before he was done bleeding, he said he was greatly relieved, but such was his imprudence that he was found by the surgeon upon deck, next day, smoking his pipe, after taking more than a moderate glass of some spirits, which checked his recovery, yet it continued slowly, until he was perfectly well, and the effects of bleeding decided indisputably in favor of further trials; but both the surgeon and his mate were taken ill very soon after this, and were in such a deranged state that they did not know the relief and benefit it afforded.

" 'The intellectual feelings of all who died were, for about twenty-four hours before their death, succeeded by a turbulent distraction of mind, and they all emitted a great quantity of blood directly before or after their last breath.

" 'It is already observed, that on Wednesday evening we took our departure from the island Tortuga. Died this day, five men and a boy.

" 'Thursday the 21st, got in sight of the island Henegar. Died four men.

" 'Friday, made the island Mayaguany, and in the evening took our departure, it being the most northerly land in our intended course. Died five men.

" 'The master (Roger Taylor) had been till now able to look after the duty of the ship; his disorder was a bilious one, and which, at this time, reduced him to the necessity of keeping his bed. Every day now increased our despair.

" 'Saturday the 23d, latitude 25° 10' N. died Captain Stevens and two men. The remains of the captain were committed to the deep with military honours, at twelve o'clock at night.

" 'Monday, died three men. This day the surgeon, after suffering long from severe head-achs, occasioned, as was thought, by want of rest, was found lying under the table of the ward-room, from whence he was brought to his cabin, where he was locked up, or attended by some of the men, to prevent him from running distracted through the ship. His indefatigable attention to the sick, as long as he was able to stand, deserves the grateful recollection of those who witnessed it; and so far was he prejudiced against bleeding, though he saw something of the good effects of it, that he would not submit to the operation.

" 'Our prospects were now very gloomy; in an immense wide ocean, the ship full of a contagious fever, deprived of every medical assistance, and also of those who were entrusted with, or in the practice of navigating, the ship.

" 'The purser (James Hatton) was the only one on board, except cept the master, (whose life was now despaired of) that understood any thing of navigation. Perhaps one who would only think of the situation we were in may say, Why did you not put back, or put into one of those islands you have passed? and, probably, one may suppose that the captain, as above, insinuated a wish to have advice on that subject, but no such thing could be thought of; for, were we to put back to Jamaica, it would look timid, or, in plain terms, be called cowardly. To put in at St. Domingo or Cuba, (which latter was on our larboard side as we passed the other) we could not expect to recover from the unfortunate state we were in, for no accommodation or comfort would be afforded us, and we could get nobody to come near us, and much less get any one to supply the place of any of those we had lost. The last islands we passed are commonly called the Turtle Islands, and are thinly inhabited by turtle-fishers only, so that there was no alternative but to proceed and trust to Providence.

" 'To supply the place of the medical gentlemen now became one of the most serious considerations; it is observed, that bleeding has, before this, been tried with apparent success, and Lieutenant Douglas, of the 85th, who understood nothing more of the profession than how to use the lancet, found himself under the necessity of undertaking the treatment of the sick. *The place allotted for them was now full, and others thought it dangerous to go near them; but it did not appear in the least that those, who were inseparably connected with the sick, were more subject to the disease than those who took every precaution possible to keep away from them. Notwithstanding the fate of the surgeon and his mate, there are a great many more instances in favor of this argument than against it, which, for the sake of brevity, we pass over.*

" 'Tuesday, 26th, latitude 28° 13′, died four men. Thirteen men, having the most unfavourable symptoms of the fever, were bled this and the preceding day.

" 'Wednesday, latitude 39° 9′, died five men. It evidently appeared that the men were, till now, prejudiced against bleeding; but seeing that all who had been bled, except two, (who had concealed their illness until the disease was too far confirmed to give way to the remedy) commenced their recovery from the first moment of the operation, they resigned themselves, with a degree of confidence, to it.

" 'Lieutenant Douglas observed some shyness in the sailors and marines, when any of them was taken ill; one of the 85th, or serjeant of marines, would come to report it, and ask if Mr. Douglas could be expected to take the trouble of bleeding him; but he took the earliest opportunity of removing their foolish ideas, and gave particular orders to the petty and non-commissioned officers, that the moment a man was seized with any of the leading symptoms of fever, he should be informed of it, and that at any hour, whether in bed, at dinner, or at breakfast, no consideration would induce him to delay his affording any assistance to a sick person, and that it was absolutely necessary to inform him on on the first appearance of the disease. This had the desired effect, for no shyness appeared afterwards, and there was hardly a night, until the fever began to disappear, but Mr. Douglas was called up three or four times; and, to the inexpressible happiness of every one, his attention was well rewarded with the recovery of all (the two before-mentioned and one other excepted) who came under his hands. It appeared evident that, if the patient had not been bled on, or very near, the first appearance of the leading symptoms of the fever, there could be but very little hopes of his recovery, and such was the ill consequence of trusting to the mode of curing by calomel, that, out of seventy-nine, whose fate had been entrusted to it, four only recovered. No fewer than sixty-five had been bled by Mr. Douglas, and so powerful was the good effects of it, that the greatest part of them would be found, the next or second day after they had undergone the operation, attending the work of the ship. They hardly felt any inconvenience from the incision after the second day at furthest. The faculty recommend large incisions, on the few occasions they agree to bleeding, but Mr. Douglas, from want of practice in that way, has been more timid, and was always careful to cut only sufficiently large to bleed freely, and, if the first did not give relief, to repeat it by drawing the same quantity; in some instances three times were found necessary. The quantity drawn at a time, from a strong able man, was half a pint.

" 'Thursday, died six men. Since we had lost the use of the master, how to supply his place in navigating the ship was a matter of very serious consideration. All were equally exposed to the fatal foe, and Mr. Hatton, seeing all his companions, with whom he had been a long time, and in many a perilous situation, taken away in so short a time, appeared to have an idea that he must very soon follow; and he often said he had no wish to live after them. Lieutenant Douglas, for some days before this, applied the most of the time he could spare from the sick to the study of the practical parts of navigation, in which he soon made a tolerable good progress; and keeping Mr. Hatton's mind a good deal engaged in explaining the most difficult parts, was of itself very useful. This way we passed the time until affairs began to take a turn. There were two other gentlemen, passengers, on board, (Lieutenant Franchisen, of the 60th, and Ensign Richard Longfield, of the 85th regiment) and thus, forming a small society of four, endeavoured at all times, when it was possible, to drive away all melancholy thoughts, and speak of the happy days we were yet to see in Old England.

" 'Friday, 29th, latitude 31° 30', died the surgeon, the boatswain, and three men. Mr. Taylor now began to get better.

" 'Saturday, died the surgeon's mate, Mr. Varley, one of the three women on board, and three men.

" 'Sunday, latitude 33° 6', died four men. The sick by this time were getting few by deaths, and some were still in a desperate state.

" 'Monday,

" 'Monday, the 1st of November, died three men. Hard gale all day and night, with rain and lightning, going our course at the rate of nine to ten knots an hour. The rage of the disease now began to abate. The hard gale, which continued for nine days, though against us, except the first day, must have greatly eradicated the disease, for now every day lessened the complaints.

" 'Tuesday, died two men, latitude 37° 55', wind squally and changed against us.

" 'Wednesday, died one man. Mr. Taylor was now able to come out of his cabin, supported by two men; his recovery was slow, but he attended his duty from this time.

" 'Thursday, strong gales continued with rain, died Mr. Stevens, master's mate, a fine lad of about thirteen years old, son to the deceased captain; he was taken ill on the last Sunday, but concealed it until the next day, when he was bled twice: he was thought to be better that night, but the next morning he was so ill that bleeding a third time was thought necessary, but to no effect.

" 'Friday, strong gales and rain. No death this day, for the first since the 16th of October, and only three men died after this, who had lingered a long time under something of the bad effects of the fever. We had a continuance of the same unpleasant stormy weather until Thursday, November 11. Latitude 42° 16'.

" 'Friday, 12th, moderate and fair, made soundings in ninety-five fathom water.

" 'Saturday, at nine, saw the land. South coast of Nova Scotia. Sounded in fifty fathoms. The men of the 85th were now suffering severely from the cold; they had no kind of bedding, but slept in a bare hammock, nothing of the kind being allowed them on embarking from Jamaica, notwithstanding that application had been made, and the captain having represented the likely ill consequence of men changing, at that time of the year, from the West Indies to the neighbourhood of Newfoundland; but they were most humanely treated by General Bowyer, upon our arrival in Halifax, where they were immediately supplied with a sufficient quantity of bed clothing, and money to buy them other warm articles. We hardly lost sight of land after this, and got into Halifax harbour on Wednesday, the 17th, when we met with so generous a reception as to make us forget our late distresses. We were put under quarantine till the 30th of this month, but were supplied with every article that would make us comfortable, from the shore; and the admiral, Sir A. Mitchell, who was there, as well as the general, regretted the necessity of keeping us so long confined from any other society.

" 'The necessary officers were now appointed to the ship by the admiral; a surgeon was the most necessary one in our present situation; though there were very few seized with any thing like the yellow fever, we had several sick, and we got a gentleman on Friday, that is, the second day after we came into harbour, well deserving the charge. After we got out of quarantine the sick were put into the Navy Hospital, which is an uncommon comfortable one, one, and where they all recovered before we sailed. We got several articles put in, particularly spars for Portsmouth Dock-yard, and left Halifax under the command of Captain Joseph Spear, on Sunday, the 16th of January. Nothing particular occurred on this passage. We arrived at Spithead, on Sunday, the 13th of February, where we performed a quarantine of four days.

" 'The Pratique master, according to his orders, got all the wearable articles belonging to the deceased officers, and took them to some distance from the anchoring places, where they were sunk." '—*Annual Register*, 1802, p. 814.

Long as this extract is, and in some respects unprofessional as it may seem, we have not had courage to shorten or alter it in a single instance. Nor have we thought it necessary to add two certificates, if they may be called such, one from Dr. Harness, and another from the surviving officers of the ship Chichester. The length and importance of the paper itself must be our apology, if any is necessary, for omitting them, and some valuable extracts which follow from Dr. Dickson's circular letter. The letter from Mr. Lind to the author is such as we should expect from the candour of the one and the merits of the other. The remainder of this part of the work is taken up with a view of the various modes of practice in the yellow fever at Jamaica, from all which it appears, as is now we conceive pretty generally admitted, that early bleeding is the only certain remedy, and that the *typhoid symptoms*, as they are sometimes termed, which occur at the close of the disease, are usually the effect of organic mischief from inflammation which could only have been prevented by a bolder practice in the beginning.

The second part of the work contains "Memoirs of the Fever which prevailed in Cadiz in 1810, illustrated with Cases and Dissections."

On this we shall first remark, that the author found the months of July, August, and September, so intensely hot as to render him extremely bilious and dyspeptic; in consequence of which, he obtained leave to proceed to Gibraltar. Here it was generally hot, but it was not till after he had resided a week that he found the yellow fever had been discovered on board some transports in the harbour, and on his return to Cadiz, in the early part of October, he learned that an alarming fever had manifested itself in that place also, and that its appearance was simultaneous with that at Gibraltar. The first case of which we have any history is, in our opinion, quite satisfactory as to the inflammatory nature of the disease, and the other parts of the history are a sufficient presumption of its non-contagious property, but we regretted much to see the juvenile ardour of the author explaining to his superior officer what would have readily suggested itself on the perusal of the facts, and what, if left to himself, that officer might have considered a discovery of his own. By this unfortunate forwardness Mr. Doughty was injured, and probably the service too. We should not have dwelt on the subject, had we not suspected from the latter part of the letter appearing in these memoirs, that time had not produced sufficient conviction on the writer's mind how much he was injuring himself without serving any useful purpose.

The letter to his Royal Highness the Duke of Kent, is unexceptionable, and ought to have produced a proper enquiry. Indeed, it is highly probable that it did, but that Mr. Doughty's opinion was not thought sufficient to bear down the authority of many senior officers.

A great number of cases follow, with dissections, all of them valuable; but, we trust, they will be found unnecessary in the present state of the enquiry, when the minds of most men are made up on the subject. On this account we omit all the remaining pages of this part of the work, which have much more of controversy in them than is necessary. We would also remark, that a case, which he relates with peculiar minuteness, succeeded, though the patient was neither blooded early nor boldly. We admit, that probably he would have recovered sooner, had the practice been more "decisive, 'till the head and precordia were relieved." But this is enough to show, that a patient may recover, though bled late, and that the apothegm from Pope, which the author has quoted from Dr. Johnson, is not of universal application—

"A little bleeding is a *dangerous thing;*
"Bleed free, or open not the vital spring."

We shall offer the following lines from the same poet—

"'Tis not enough your council should be true,
"Blunt truths more mischief than nice falsehoods do;
"Men must be taught as if you taught them not,
"And things unknown proposed as things forgot."

In these last remarks, we have invaded the concluding part of the book, which professes only a *recapitulation*, but contains much miscellaneous matter, all tending to confirm the great objects of the work.

We have given a very free opinion of this performance, and it will be found our remarks, or at least our objections, relate more to the *morale* than to the practice.

There is one other subject, which, as reviewers, we conceive it our particular duty to notice. We admit, that "in medical discussions, matter more than manner, should be the point in view." Yet there is a certain correctness which is not only ornamental, but necessary; and a just attention to which, in a particular manner marks a correctness in the mind of the writer. The objections we shall make, it is true, are neither numerous nor of the first importance; but we think it our duty to make them for the benefit of science, and, perhaps, because our omission might be construed into inattention.

In page 2, we meet with the "morbid nature of a cause." Now, morbid and diseased are the same, but we have no proof that the *cause* of yellow fever is *morbid: Morbific*, we conceive a more proper expression, and the more so, inasmuch as we have no English primitive word to express the sense implied. The same objection occurs at page 10, and in some other places. Among writers not professedly medical, we ought not to object to terms almost allegorical, because, for want of technical knowledge, they cannot be expected to make themselves understood by any other means; but, in medical writing, we never shall admit such language as "soils for and *seeds* of a disease," which the author in the next paragraph transforms into *sparks*.

"Yellow fever (says he) cannot be propagated in a soil which does not in itself impart the seeds of the disease.

"From this statement it may be clearly seen, that I do not consider it necessary to place a ship under quarantine because she has sailed from a port where yellow fever prevailed at the time of her departure; or that the disease even prevailed amongst the crew on her voyage. Every *spark* of that malady would be long extinct ere she reached the coast of Europe—'*Lateat scintillula forsan*,' so well considered by the Humane Society, in cases of suspended animation, would not apply to the *miasma* of yellow fever, on the principle of being diffused in our climate."—Then comes again the allusion to vegetation: "Not a *germ*, we are told, of that invisible *agent* of febrile—." To adopt once more the author's fondness for quotation, we should say,

"Nec quot confundis aut jura aut nomina sentis."

These, however, are unimportant objections, compared with the merit of the work; but that very merit makes us wish to see it without such blemishes.

### *Medico-Chirurgical Transactions, published by the Medical and Chirurgical Society of London.* Vol. VI.

(Continued from p. 70.)

### *Observations on the Mediterranean Fever;* by ALEXANDER DENMARK, M.D. &c.

WE know not whether our readers are tired of fever. But, if we may judge by ourselves, the subject cannot be examined with too much minuteness, and the multiplicity of evidence is the only means by which we are to judge of the comparative accuracy of each. Dr. Denmark's paper is evidently written only with a view to confirm the now generally received doctrine, as far as it is applicable to practice. It contains, however, too many good things for us to pass over. First, the author's arch objection or sly fling at nosology.

"It does not (says he) much matter whether we look upon fever as an idiopathic or symptomatic disease; whether we regard it as occasioning topical congestions and inflammations, or these as giving rise to fever. Our opinions on these points may, indeed, derange our nosology, but, fortunately for our patient, they cannot materially affect our practice. They may tend to throw us off our guard with respect to infection; but the grand outline of our treatment must remain the same. Neither shall we insist upon giving exclusive credit to the brain, the liver, the stomach, nor any other viscus, either for bearing the brunt in all the patient's sufferings, or for disseminating so much mischief to the machine at large. For I believe the fact to be undeniable, that all these viscera have been found, at different times, to be individually, and sometimes collectively, affected, in the very same fever.

"The names of Boyle and Irvine, I believe, stand foremost in establishing the decisive superiority of the treatment of the Mediterranean fever, by depletion."

Highly as we respect the above names, we cannot forget the obligations we owe to the venerable Cleghorn; and, if the Cullenian practice unfortunately superseded for a time all the benefits we should have derived from the physician of Minorca, we ought not to forget that the persecuted Jackson was among the earliest in resorting to the free use of the lancet in most of the fevers which occur in the service, from whatever cause and in whatever climate.

Another observation struck us still more forcibly, as confirming the practice of the same gentleman, and explaining, in our opinion, a passage in Celsus, which, for want of being well understood, has proved a source of unnecessary dread, and, we fear, of erroneous practice.

"Those of the ship's companies (says Dr. D.) seldom failed to complain on the occurrence of the first symptom of indisposition;

 and, and, as I had requested that they might be sent on shore as soon after their becoming ill as possible, they were received at the hospital, in many instances, before the termination of the chilliness and shivering which ushered in the fever. In these instances, venæsection was never had recourse to till the vascular system had fairly emerged from the depressed state incident to that stage of the fever, and re-action had clearly manifested itself by the returning glow of the skin, the filling of the previously shrunk and dejected features, and the firm, though frequently oppressed, beat of the pulse. Former experience not only taught me that an earlier abstraction of blood was never borne to an extent productive of ultimate benefit; but, on the contrary, seemed to be injurious, by tending to protract the first stage of the paroxysm. I am afraid that due regard has not been paid to this circumstance; and that, in the recent rage for phlebotomy, it has been too much overlooked. I am, indeed, induced to ascribe to this oversight consequences replete with still more serious evils, for, where persons have witnessed the loss of a few ounces of blood to be borne so badly, they have sometimes inferred, without looking to the real cause, that the disease was one of *debility*. The treatment was, of course, adapted to the diagnosis."

Now, it is well known that in many instances Dr. Jackson found it necessary to precede his blood-letting by warm bathing, and many other comforts, after which he was enabled to form his "diagnosis, and to adapt his treatment." Celsus has a passage—"Si vehemens febris urget, in *ipso impetu ejus* sanguinem detrahere, hominem jugulare est." Lib. 2. cap. 10. In the context he advises blood to be taken on the second or the third day of a fever. But, adds he, though it may be sometimes necessary to bleed on the first day, yet it never can be useful to bleed after the fourth. By the *ipso impetu*, therefore, may we not conceive he meant the cold stage, or *crude state*, to use the, unfortunately in this respect uncertain, language of Celsus and Sydenham.

The above explanation is offered with some diffidence, but we cannot help conceiving it confirmed when Celsus admits bleeding sometimes on the first day; and still more when we connect it with a preceding precept, in which he seems, in a particular manner, to describe the beginning of a hot stage. "Ergo vehemens febris, ubi rubet corpus plenæque venæ tument, sanguinis detractionem requirit." We shall, therefore, conclude these remarks with showing a further coincidence in the practice of Celsus with that of the reformed moderns, in nearly the same latitude. The caution of Celsus is—"sanguinem mittere post diem quartam nunquam utile est, cum jam spacio ipso materia vel exhausta est, vel corpus corrupit, ut detractio imbecellum facere possit non possit integrum." Dr. Denmark's words are, "The danger danger consists in either applying the remedy too late or too often; and it has fallen to my lot to see, under my own direction, the abstraction of blood accelerate the patient's death, and this too under circumstances that would fully justify its employment. After the supervention of the yellow suffusion, whatever may be the other symptoms demanding it, venæsection, I believe, will not be borne with impunity." P. 305.

The coincidence of these two is remarkable, and the modesty of the latter is not less praiseworthy. But to us it appears that the writer might spare himself any compunction, for we believe what Celsus seems to hint, that neither bleeding nor any other remedy could relieve these cases, and that they die, not from the loss of blood, however unnecessary or improper in that advanced stage of fever, but from an altered state of some important viscus. Of this Celsus had no means of judging, and Dr. Denmark regrets that his own opportunities were so few.

We shall not presume to offer any opinion on the other remedies, not only because the author, from his own experience, must be the fittest judge, but because, from all we have seen of this practice, if blood is freely drawn in the beginning, the rest of the treatment will be easily determined by the various indications.

*Experiments and Observations in order to ascertain the Means employed by the Animal Economy in the Formation of Bone.* By John Howship, Esq.

The name of Mr. Howship must be familiar to our readers, from the many valuable communications with which he has honoured us. They will, therefore, anticipate much interesting information when a writer, so accurate in his description of morbid appearances, directs his attention to physiology. In this they will not be disappointed.

Mr. H. commenced his inquiries in the human fœtus. An embryo, eight weeks old, was prepared, by spreading out the limbs on slips of glass, allowing them to dry, and afterwards examining them by an instrument constructed on the principle of the solar microscope. We would here stop to inquire whether the apparatus of the common magic lanthorn might not answer all the purposes, and save the trouble and expense of constructing any thing fresh?

On a careful examination of his subject, it was discovered that bony rings had been formed in the situation of the metacarpal bones, and of the first and third phalanges. The diameters of these bones was much larger in proportion than at a more advanced age. This was most evident in the hand and and foot, and appeared to be a provision for admitting a considerable increase to the length of the cylinder before it became necessary to enlarge the diameter. At this time no cartilage could be discovered in the situation of the joints, which consisted only of a yellowish transparent gummy matter.

In an embryo ten weeks old, the extremities of the bones were found connected together by a cartilaginous substance: the rings described in the former examination were now gradually increased in length, and had reached the cartilaginous portions at the extremities.

An embryo thirteen weeks old was injected with size and vermilion. The cavities within the cartilages, as well as the cancellated part of the bone, received the injection.—A fœtus of seven months was most successfully injected. The cartilage of the thigh bone had acquired a comparative firmness in its structure. All the cavities were formed into canals, traversing the substance of the cartilage.

From these and some other examinations, it was ascertained that the first and earliest state in which the particles of ossific matter become apparent, after they have formed a mass by their cohesion, consists of delicate fibres, forming short parallel tubes, and opening externally on the surface connected with the cartilage.

"In order to observe the changes that occur towards the latter periods of growth, sections were taken from the lower end of the thigh-bone; these were selected from subjects of various ages, and the following were the appearances under the microscope.

"In a child' eleven months old, the canals within the cartilage were very few in number. At the age of four years, these canals were still more thinly scattered, and those that were observed were of comparatively small diameter. When the sections became partially dry, a line one-sixteenth of an inch in breadth was seen towards the margin of ossification, where the particles of the cartilage had apparently taken on a new arrangement, so as to resemble parallel lines or fibres. This curious circumstance has been noticed by Haller.

"At the age of eleven years, the cartilaginous canals were found to be still diminishing, both in point of size and number; and in the examinations made at seventeen years, it was with great difficulty that a section could be found in which there was any remaining trace of them."

The writer's next examinations were in quadrupeds. Here, as might be expected, he found little difference from the human. These slight differences, however, are marked with philosophic accuracy. His next inquiries were in the larger mammalia, viz. the cetaceous tribe, and afterwards on birds.

A second section of the paper follows, on the formation of the the bones of the head. In the examination of these, at various ages of the fœtus, particular attention is paid to the subject, as an opake as well as transparent object. The result is extremely interesting, particularly the description of a gelatinous fluid deposited between the scalp and the skull, and separating them to the distance of a quarter of an inch.

"This jelly was transparent, but had a slightly red tinge, and was readily removed from its situation. Laid upon glass, it very soon lost a part of its bulk, by the oozing of a clear limpid fluid. Upon placing this mass under the microscope, it was found to be beautifully and most abundantly vascular. A numerous assemblage of the finest capillary arteries, very variously inflected, was suspended in this bed of transparent matter.

"Seeing that this substance lay in immediate contact with the bones of the cranium, and was furnished with a vascular arrangement peculiar to itself, there could no longer be any doubt of its being the loose cellular state of the fœtal pericranium, loaded with a serous exudation from the vessels in consequence of their being injected; although the greatest magnifying power afforded no distinct trace of cellular web or membranous structure.

"By way of experiment, a part of this vascular jelly was laid upon a slip of glass, and exposed to a gentle heat: an aqueous vapour soon arose, and continued to pass off, till at length the gelatin assumed the form of a dried membrane. On examination under the microscope, it was found, contrary to expectation, that the brilliancy of the injected vessels, which it had been supposed would be obscured, was not diminished by the evaporation of the moisture.

"The dura mater was next examined, and, as I had just been contemplating the extensive anastomosis and continual inflexions of the vessels on the outside of the skull, I was forcibly struck with the contrast in the appearance of the arteries destined to circulate the blood on the inside of the same fabric."

The conclusions drawn by Mr. Howship are as follow:

"1. That, in the mammalia, the first rudiments of ossification in the long bones are the effect of a secretihg power in the arteries, upon the internal surface of the periosteum, which produce a portion of a hollow cylinder; this form of bone having been found antecedent to the evolution of any cartilaginous structure.

"2. That, at a certain stage of the process, the mode of operating is changed, in order that it may proceed more expeditiously. A cartilage is formed, which, by the nature of its organization, and by admitting of a specific provision of cavities and canals lined with vascular membranes, which secrete an abundant store of gelatinous matter, is adapted to this particular purpose; while at the same time it serves to determine the future figure of the extremity of the bone, by establishing and conducting the ossification, within its own substance.

"3. That, from the appearance and texture of cartilage, when examined examined under the microscope, it may be defined,—an even and finely granulated albuminous matter, deposited in the interstitial spaces of an exceedingly elastic bed of a semi-transparent reticulated structure, which is apparently a modification of gelatin.

"4. That, from the period when the ossification proceeds in the mode above described, by the medium of cartilage, the process is continued in the same uniform manner till it has completed the growth of the bone. The growth of the epiphyses, and their union with the ends of the bone, are also affected by the same means.

"5. That the ossific matter in the cylindrical bones is deposited primarily in the form of fine thin tubular plates: a mode of deposition of all others the most favourable for their being subsequently re-modelled, and for facilitating all the subsequent changes of structure they are destined to undergo.

"6. That while the circulation in the capillary arteries situated between the cartilage and bone must provide the phosphate of lime, the principal agent in extending the cylinder, and in effecting the subsequent progressive changes of structure, which in a growing bone are continually taking place, appears to be simply the mechanical pressure exerted by the fluid secretions within the medullary cavities of bone, this power operating successively in different directions, according to the particular determination given by the circulation.*

"7. That the mode of circulation most favourable for ossific action, is a very slow and uniform motion of the blood through the capillary system; and that the numerous inflections of the minute arteries in the pericranium, and the great weakness, and rectangular mode of giving off the smaller arteries upon the dura mater, as well as the extremely curious appearance of the blood and injected matter, upon the fine membranous linings of the canals in cartilage, indicating, as I believe, something beyond a mere capillary circulation, are to be considered as so many evident provisions for securing this condition.

"8. That, in the formation of the cylindrical bones, the ossific surface is arranged into tubular plates of two different sizes, constituting a larger and a smaller series; an arrangement by no means essential to the increase of a bone, because, in many of the early stages of ossification, and also where the growth is very slow, the larger series is found to be entirely wanting.

"9. That the only apparent use of the larger series of tubes, is that of augmenting the quantity of blood circulated through the ossifying structure, so as to increase the rapidity of growth; for they are abundant in animals of quick growth, less numerous in

---

"* This opinion, however extensively it may bear, will not appear strained to those who will for a moment consider the general incompressibility of fluids."

those that reach maturity slowly, and in the same animal I have observed they are employed by nature, or laid aside, in conformity with the quick or slow developement of structure, which we know actually takes place at the particular period when the examination is made.

"10. That, in the growth of the cylindrical bones, and of those flat bones that are formed upon cartilage, the deposit of the ossific secretion is in the first instance made around the external openings of the smaller series of tubes, and upon these only. This opinion derives support from the recent appearances of the bones of quadrupeds, but is most clearly established by the characters found upon the ossific surface in the bones of birds, where the gradations of progressive evolution are more readily traced.

"11. That, in the flat bones of the skull, the circumstances under which ossification takes place, differ materially from those above described. In these the phosphat of lime in combination with the animal mucilage, is occasionally deposited in small detached unequal masses, without regularity, as if merely laid in the way, preparatory to their subsequent application; that these soon become connected with the more central parts of the bone, and are found to decrease in thickness, as they increase in breadth, until they are finally consolidated with the original plate of bone.

"12. That the particular simplicity observable in the mode of production of the bones of the skull, affords a strong argument in favour of the opinion, that pressure, variously modified, constitutes one of the most efficient instruments in the hand of nature: for, in this instance, the uniform though gentle pressure from the impulse of the circulation, and the constantly increasing volume of contents in the head, must be admitted to be the sole agents in completing that process, which, in its commencement, had the appearance of being conducted in a comparatively imperfect manner.

"13. That the ultimate texture of bone is not laminated, but reticulated, the phosphat of lime being deposited as an interstitial substance; for, although from the greater compactness necessary to the bones of quadrupeds, the ultimate structure is not in them so readily traced, yet, in the more delicately constructed bones of birds, this mode of arrangement is sufficiently obvious, and may at any time be readily ascertained."

Mr. Howship, with much propriety, remarks, that many of the above observations and opinions have been in part confirmed by other physiologists. Among the rest, we think the late Mr. Gibson, of Manchester, ought not to have been omitted. We are far, however, from imputing this omission to any intention of the author, who, throughout the whole, evinces equal modesty, ingenuity, and correctness. The paper is illustrated with coloured engravings.

EMMERT's (Prof. Med. at Bern) *Observations upon the Poisonous Effects of the spurious Cortex Angustura.*

[From our German Correspondent.]

THE first intelligence of the distinguishing traits of the different sorts of the Cortex Angustura, occurring in trade, and the first publication of the poisonous effects of the spurious or bitter East-India Angustura, we owe to Dr. Rambach. The Austrian government, on occasion of a case of poisoning with the bitter Angustura occurring in Hungary in 1806, was induced to subject the different sorts of Angustura to a closer investigation, when it was found that the druggists' shops contained three different kinds:

1. Commonly used in the apothecaries' shops, is a thin smooth bark, of a yellowish colour in the fracture, and of a bitter aromatic taste.

2. Is a thick orange-coloured spotted bark, in the fracture appearing on the outer edge of a yellowish white, and on the inner of a brownish colour; of an unpleasant bitter, not aromatic, taste.

3. A less thick, but white or yellowish white, bark, in the fracture dark grey, on the inner edge yellowish, partly approaching to brown, of an unpleasant bitter taste, and hardly possessing any aroma.

From experiments made upon dogs, it appeared that No. 2 and 3 produced mortal effects; but No. 1 might be given without detriment.

However, these experiments were still imperfect, neither explaining the circumstances under which the bitter bark proves poisonous, nor the accidents which it produced.

The merit of Prof. Emmert is, of course, so much the greater, as, by his experiments, the results of which we shall communicate to our readers, he has roused the medical authorities from that indifference with which this article has hitherto been treated.

Mr. E. made his experiments with the spurious or poisonous Angustura, with equal success, on mammalia, birds, amphibia, and fishes. He, for the most part, employed a concentrated decoction, and exposed to its operation the membranæ mucosæ, membranæ serosæ, the epidermis, the inner surface of the same, the muscles, tendons, and nerves.

Thirty experiments, made with great care, yielded the following results:

1. The spurious bitter Angustura is a violent poison for animals of the four superior classes.

2. The poisonous effects first show themselves in parts of the body provided with a great number of blood-vessels, or with larger blood-vessels vessels under a thin covering, such as the pleura and peritonæum; but cannot affect the body, in its peculiar manner, either from the uninjured epidermis, nor the nerves and sinews.

3. It affects the different animals, and the different organs of the same animals, in a similar manner.

The symptoms produced are, a difficult, at first accelerated, breathing; a quick, afterwards convulsive, pulse; anxiety; a lessened command of the muscles, particularly of the hinder extremities; spasmodic stiffness of the limbs, sometimes also of the breast; tremor; startings, as if of electrical concussions, particularly along the columna vertebralis, which take place either on a strong or gentle, and scarce perceptible, impression made, or sometimes spontaneously; tetanus, mostly in form of opisthotonus in its most violent form; the opening of the eye-lids and the pupils are enlarged to the utmost; the eyes, fixed and immoveable, start from their sockets; the lower teeth closely pressed against the upper; the pulse intermittent, small, and convulsive; the respiration ceases entirely; and those parts, otherwise red, now assume a blue colour, and not a single organ of the body shows any re-agency against the irritations applied to it. In about a quarter or two minutes at farthest, the attack of tetanus subsides, the pupils and eye-lids contract, the eyes return again into their sockets, the body grows relaxed and flaccid, respiration impetuously and with difficulty returns, becomes gradually more free, but still continues laborious; the arterial blood becomes darker, the pulse more frequent, fuller, and more free in general, but still a little strained and hard. The irritable condition of the body, the want of power in moving the limbs, and a certain stiffness in them, still continues; the electrical shocks, and tetanus, are excited by the least impression on any sensible part of the body, and the nervous attacks return spontaneously after longer or shorter intervals. If the animal recovers, the returns are less frequent, and milder; but, in the contrary case, the animal rarely recovers.

The sensibility seems to be exalted, and the terror also, as in hydrophobia; for all the animals labouring under the effects of the bitter Angustura are sensible of the slightest impression made on their organs of hearing, sight, and feeling, and start fearfully.

Perceptible evacuations do not attend this process of poisoning: vomiting never took place, except in a crow, that vomited pills mixed with the bitter Angustura; and stools only occurred when the same was introduced in form of a glyster. After apparent death, neither the irritability of the muscles, nor the sensibility of the nerves, were, at first,

 lessened; lessened; only the stiffness observed in dead bodies took place at an early period.

The general affection of the animal body, by this poison, seldom takes place before the seventh minute. Till this period, the animals are lively, and even feed with appetite. Sometimes the first attack of the tetanus ends in death, but commonly in the succeeding one, after two, three, or five minutes; but also, in some cases, after some hours. This depends on the difference in the parts to which it is applied, and the dose given. The reconvalescence from this poison proceeds but slowly, and the hinder extremities recover last of all.

The poisonous effect proceeds much more violent and speedy on the serous membranas and open wounds, than by the intestines. On being introduced into the blood-vessels, its effect is quickest and strongest. Neither coffee, vinegar, nor oil of turpentine, stop the poisonous effect after a deadly dose; nor is its general influence removed by isolating, as much as possible, the infected parts from the nervous system, or separating the medulla oblongata; yet the latter operation enables the animal longer to resist the operation of this poison. Like many other poisons, such as the viper's, arsenic, opium, prussic acid, tartarus stibiatus, the bitter Angustura can only spread its poisonous effects from the single parts over the whole body, by penetrating immediately into the blood-vessels belonging to these parts.

We may finally conclude, that the bitter Angustura produces the same effects on the human body, as, from these experiments, it appears to have done on animals; as not only the accidents following its application in Hamburgh and Hungary speak for it, but also because most poisons affect both mankind and animals in a similar manner, as is the case with the Nux vomica, which, in every respect, coincides with the spurious Angustura.

---

